# The mitochondria-targeted Kaempferol nanoparticle ameliorates severe acute pancreatitis

**DOI:** 10.1186/s12951-024-02439-y

**Published:** 2024-04-03

**Authors:** E Wen, Yi Cao, Shiwen He, Yuezhou Zhang, Lanlan You, Tingqiu Wang, Zhigang Wang, Jun He, Yi Feng

**Affiliations:** 1 Department of Ultrasound, The Second Affiliated Hospital of Chongqing Medical University, No 76, Linjiang road, Chongqing, China; 2 Precision Medicine Center, The Second Affiliated Hospital of Chongqing Medical University, Chongqing, China; 3 Department of Hepatobiliary Surgery, The Second Affiliated Hospital of Chongqing Medical University, Chongqing, China; 4 The First Affiliated Hospital of Chengdu Medical College, No.278, Baoguang Avenue, Xindu District, Chengdu, 610500 Sichuan China; 5 Institute of Burn Research, State Key Lab of Trauma, Burn and Combined Injury, Chongqing Key Laboratory for Disease Proteomics, Southwest Hospital, Third Military Medical University (Army Medical University), No 76, Linjiang road, Chongqing, China

**Keywords:** Kaempferol, TK bond, Nanosystem, Mitochondrial homeostasis, Severe acute pancreatitis

## Abstract

Kaempferol (KA), an natural antioxidant of traditional Chinese medicine (TCM), is extensively used as the primary treatment for inflammatory digestive diseases with impaired redox homeostasis. Severe acute pancreatitis (SAP) was exacerbated by mitochondrial dysfunction and abundant ROS, which highlights the role of antioxidants in targeting mitochondrial function. However, low bioavailability and high dosage of KA leading to unavoidable side effects limits clinical transformation. The mechanisms of KA with poor bioavailability largely unexplored, hindering development of the efficient strategies to maximizing the medicinal effects of KA. Here, we engineered a novel thioketals (TK)-modified based on DSPE-PEG2000 liposomal codelivery system for improving bioavailability and avoiding side effects (denotes as DSPE-TK-PEG2000-KA, DTM@KA NPs). We demonstrated that the liposome exerts profound impacts on damaging intracellular redox homeostasis by reducing GSH depletion and activating Nrf2, which synergizes with KA to reinforce the inhibition of inadequate fission, excessive mitochondrial fusion and impaired mitophagy resulting in inflammation and apoptosis; and then, the restored mitochondrial homeostasis strengthens ATP supply for PAC renovation and homeostasis. Interestingly, TK bond was proved as the main functional structure to improve the above efficacy of KA compared with the absence of TK bond. Most importantly, DTM@KA NPs obviously suppresses PAC death with negligible side effects in vitro and vivo. Mechanismly, DTM@KA NPs facilitated STAT6-regulated mitochondrial precursor proteins transport via interacting with TOM20 to further promote Drp1-dependent fission and Pink1/Parkin-regulated mitophagy with enhanced lysosomal degradation for removing damaged mitochondria in PAC and then reduce inflammation and apoptosis. Generally, DTM@KA NPs synergistically improved mitochondrial homeostasis, redox homeostasis, energy metabolism and inflammation response via regulating TOM20-STAT6-Drp1 signaling and promoting mitophagy in SAP. Consequently, such a TCM’s active ingredients-based nanomedicine strategy is be expected to be an innovative approach for SAP therapy.

**Graphical Abstract:**

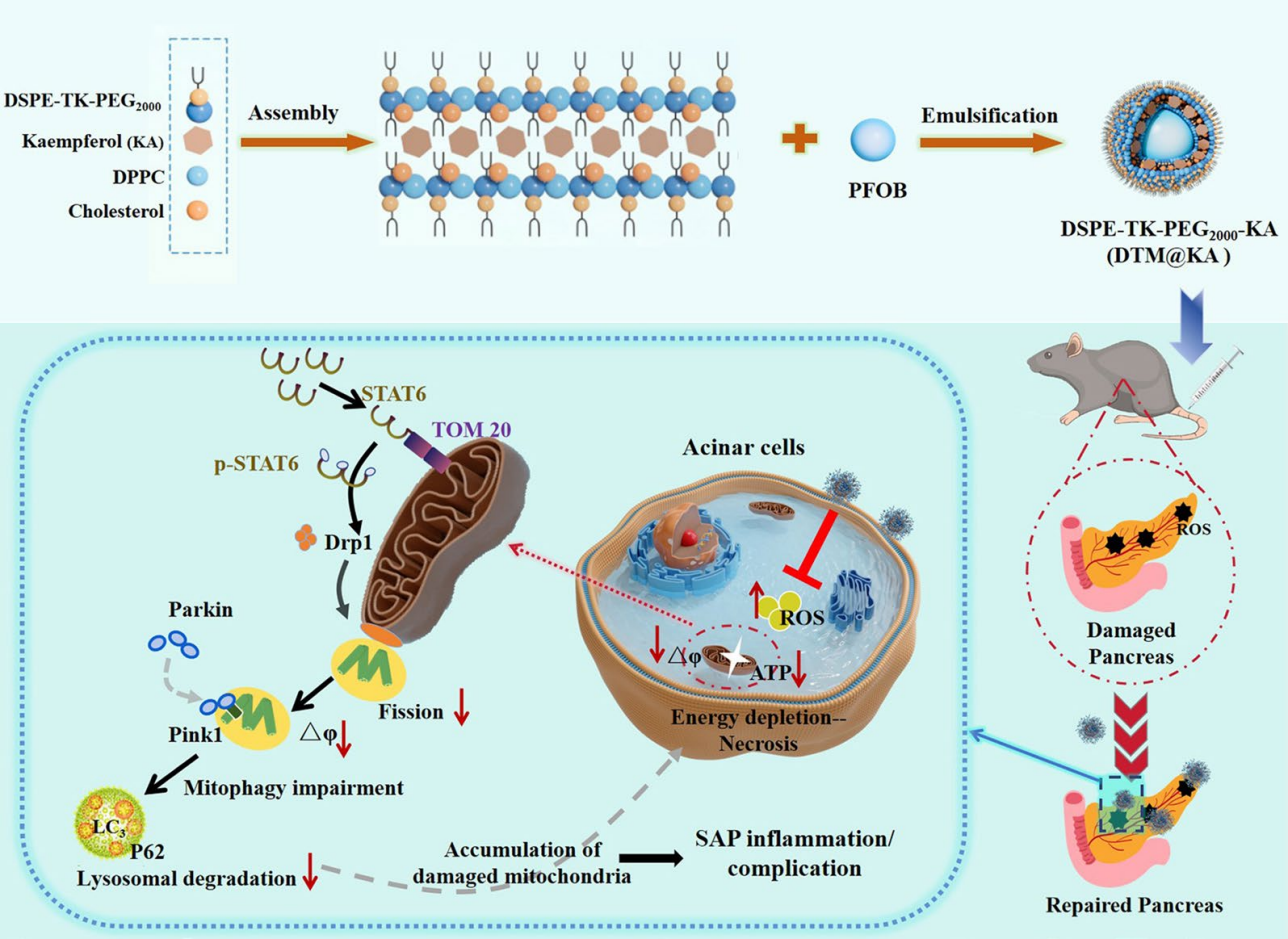

**Supplementary Information:**

The online version contains supplementary material available at 10.1186/s12951-024-02439-y.

## Introduction

Severe acute pancreatitis (SAP) is a common and fatal digestive inflammatory disease with ATP depletion caused by mitochondrial dysfunction, which is regarded as a central event in SAP, but, still lacks efficient treatments for this condition [1]. Current clinical therapy is still limited to fluid supplementation and supportive care and disordered immunity or other organs damage in SAP impede the therapy efficiency of conventional drugs [2], so combination of multiple treatments has been advocated for SAP therapy owing to synergistic activity for increasing efficacy. The chemotherapeutic drug by virtue of nanosystem delivery into targeting cell for enhancing synergistic efficacy has been gaining more attentions. Notably, natural effect components (NEC) of traditional Chinese medicine (TCM) delivered by various nanocarriers including liposomes has been gradually used to treat SAP [3]. However, TCM’s active constituents-loaded dual-delivery system with safe and efficient dual-delivery system, designed with appropriate administration for successfully delivering payloads to the target sites, remains an enormous challenge.

Mitochondrial homeostasis is inextricably linked to energy metabolism and REDOX homeostasis, which is crucial to exerting multiple biological roles of pancreas [4]. It has been well accepted that mitochondrial dysfunction contributes significantly to the inflammatory pathogenesis of SAP, including the impaired energy production, ROS generation, inflammation and pancreatic damage [5]. Extensive evidence indicates that the disrupted mitochondrial dynamics exerts profound effect on initiating and exacerbating pancreatitis in both clinical and experimental studies [6]. Stable fission, fusion and precursor protein transports in mitochondria are essential for mitochondrial biogenesis and functioning. Additionally, mitophagy is nonnegligible for removing damaged mitochondria and factors. Therefore, sustaining the proper mitochondria homeostasis offers a strategic foundation for promising therapeutic approaches for SAP.

Kaempferol (KA), one of the most representative components of flavonoids in TCM, is a favourable natural antioxidant concerned widely as a potential therapy for inflammation [7]. Numerous researches reported that KA displays an important therapeutic potential in suppression of mitochondrial impairment and anti-inflammation [8], which is conducive to be used in pancreatitis, gastritis or other abdominal inflammation diseases. In light of these, KA has been considered to be clinical medicine and food health remedies due to its multiple pharmacological properties [9]. Nevertheless, the therapeutic efficiency is largely restricted due to KA’s poor lipid solubility and water solubility. Moreover, KA is easy to be oxidized with its poor stability. Numerous advanced nanoagents modifying monomer of TCM with minimal adverse effects have been explored to underpin a solid foundation for improving anti-inflammation efficacy. Hence, KA remedied with the optimized nanotechnology may appear to be more implementable and meaningful.

**Scheme 1:**
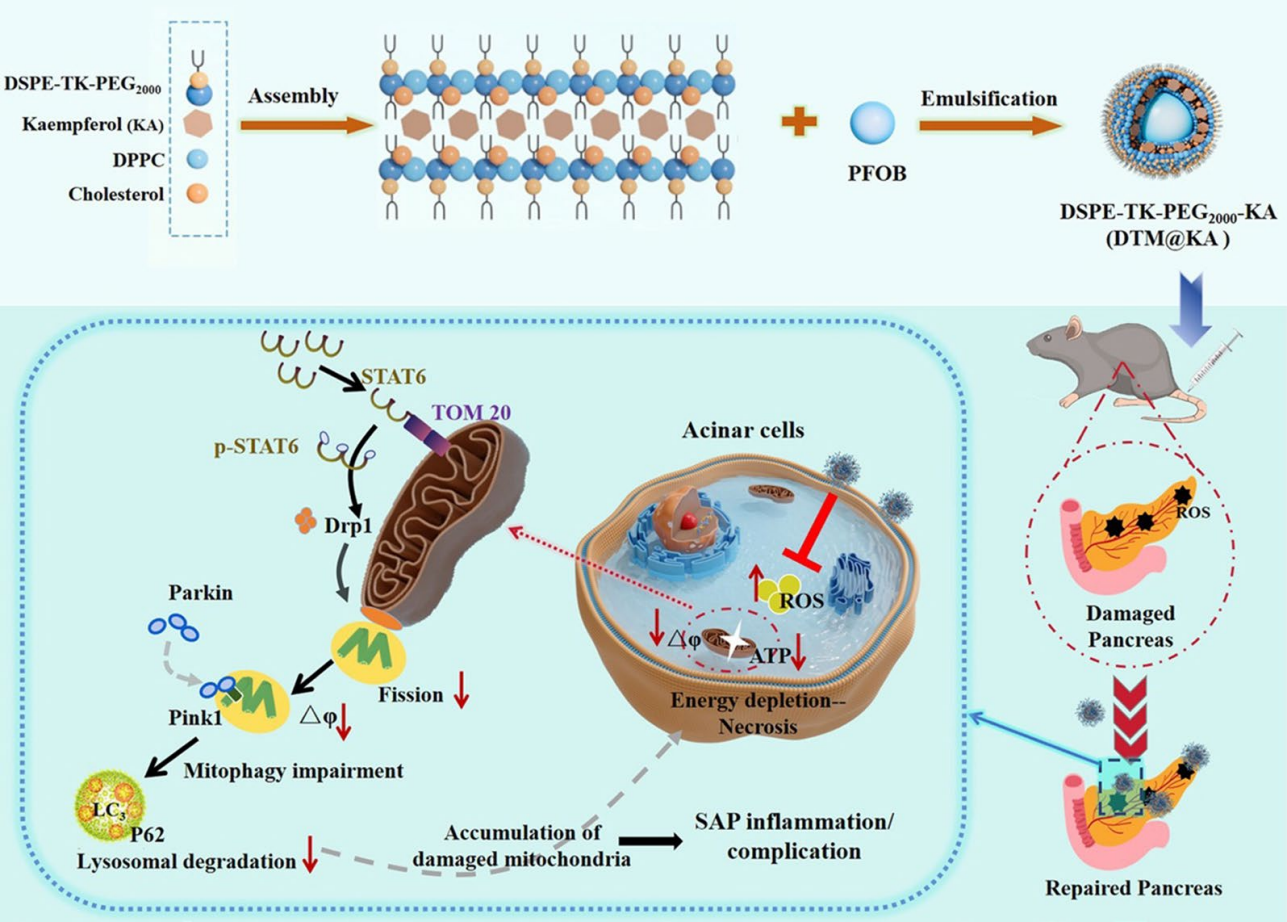
Schematic of study on DTM@KA NPs preparation and protective function as well as possible mechanism about mitochondrial function and oxidative stress regulated by TOM20-STAT6-Drp1-mitophagy signaling in experimental SAP

It has been well accepted that the impact on the mitochondrial function may be the main way KA works in alleviating oxidative stress and apoptosis [10]. ROS-responsive nanomaterials delivery system is implicated in directly and effectively treating pancreatic disease via mediating mitochondrial function [11]. KA loaded in appropriate liposomes would exert maximum antioxidant and anti-inflammatory effects after overcoming its own defects [12]. Hence, KA was utilized by us to engineer a multifunctional nanomedicine (denoted as DSPE-TK-MPEG2000-KA, DTM@KA NPs) regulating mitochondrial homeostasis for anti-oxidation and anti-inflammation via improving mitochondrial fission and mitophagy (Scheme 1).

In this study, we designed a mitochondrial function-restoring nanosystem loaded with KA based on a ROS-responsive nanoparticles, which is characterized by thioketals (TK) bond inserted into DSPE-MPEG2000 structure with improved solubility and bioavailability. TK would break liposome’s morphological integrity to release effector substance once oxidation stress [13], contributing to enhancing drug’s efficiency via targeting delivery to tissue or cells. Upon the breakage of TK bonds, nanoparticle decomposes and releases KA targeting and accumulating at sites of oxidative damage in the pancreas for longer time. After DTM@KA NPs administration, the mitochondrial damage and ROS accumulation were inhibited by balancing mitochondrial fission and mitophagy to clear the damaged mitochondria. Significantly, DTM@KA NPs was proved to activate signal transducer and activator of transcription 6 (STAT6), which improves Drp1-deficient fission with the accumulation of damaged mitochondrial and mitophagy impairment via interacting with mitochondrial translocase of the outer membrane 20 (TOM20). Such an engineered mitochondria-improving nano-liposome based on a NEC offers a feasible pathway for SAP treatment and embodies a promising potential in clinical translation.

## Materials and methods

### Regents and chemicals

KA and Sodium taurocholate (NAT) was obtained form MedChemExpress (New Jersey, USA). DSPE and PEG_2000_ were purchased from RuixiBio (Xi’an, China). DIR iodide was offered by Bioss (Beijing, China). Myeloperoxidase (MPO) and Malondialdehyde (MDA) assay kit were acquired from Nanjing Jiancheng Bioengineering Institute (Nanjing, China). Mouse IL-1β ELISA Kit and total superoxide dismutase assay Kit were form Beyotime Biotech (Shanghai, China). Antibodies against Bax and Bcl-2 were provided by Proteintech (Wuhan, China). Nrf2 and HO-1were supplied by Bioss (Beijing, China). Caspase3 and Cleaved-caspase3 were products of Abcam (USA). The SPN-9001 Histostain^™^-SP kits was supplied by Zhongshan Jinqiao Biotechnology Co., LTD (Beijing, China). All other chemicals were purchased from Sigma-Aldrich (St. Louis, MO, USA), if not otherwise stated. All chemicals and reagents were purchased commercially and were used as the manufacturer's instructions.

### Preparation of DTP@KA

On the basis of the previous studies [14], 2.0 g acetone solution (11.08 mmol), 1.78 g 3-mercaptopropionic acid (16.8 mmol) and catalytic tallow fatty acid, which all are stirred for 12 h at 25 ℃ under a nitrogen atmosphere, crystallized on ice to obtain the precipitate, and then filtrated, washed by hexane and cold water, finally dried for obtaining TK powder. Next, 7 ml anhydrous dimethyl sulfoxide was used for dissolving 39.0 mg TK and 22.6 mg 4-dimethylaminopyridine to obtain TK solution, and then 190.5 mg dicyclohexyl carbodiimide dissolved by 3 ml anhydrous dimethyl sulfoxide was slowly added and stirred at 60 °C to produce the mixture after 1 h, which was followed by being added 115.5 mg DSPE2000 (dissolved in 3 ml anhydrous DMSO). All the above reaction lasted for 24 h at 60 °C and under a nitrogen atmosphere. On the other side, 3 ml anhydrous DMSO was added to dissolve 309 mg PEG2000 and then incubated for 24 h to obtain the precipitate, which next was dissolved and then dialyzed with deionized water. Finally, the DTP was obtained after being freeze and dried under vacuum for 12 h.

Next, according to previous reports [15], 6 mg DPPC, 2 mg cholesterol, 2 mg DTP and 1 mg KA were fully dissolved by 15 ml chloroformic solution, which was steamed by the rotary evaporator ((Yarong Inc, Shanghai, China) with 80 rpm and at 50 ℃ for 1 h to form a lipid film at the bottom of the round-bottomed flask. And the lipid membrane was hydrated with 4 ml PBS and completely dissolved after oscillating on the shaker for 1 h. And then a sonicator (Sonics & Materials Inc., USA) was used for emulsifying the suspension at 100W (5 s on and 5 s off) after adding 200 μl PFOB into. The emulsified solution was centrifuged at 8000 rpm for 3 times and 5 min each time, and then re-suspended with 1 ml PBS buffer. Therefore, DTP@KA NPs liposome was prepared and stored at 4 ℃.

### Characterization

The optical microscope and transmission electron microscope (TEM; Hitachi-7500, Japan) were used for observing morphology and size of the NPs. The Zetasizer Ultra (NanoBrook Omni, Brookhaven Instrument Ltd, UK) was performed for measuring particle size and surface potential; and the liposome was placed at 4 ℃, the morphology and size changes of 7 consecutive days were observed under an optical microscope. The ultraviolet spectrometer (UV-3600, Shimadzu, Japan) was used to determine ultraviolet absorption spectrum and KA content via calculating encapsulation efficiency and drug loading capacity [16].

### ROS-responsive performances

H_2_O_2_ solution with different concentrations of 1, 10, 100 μM was prepared, and it was taken at 0, 1, 3, 5, 7, 9 h after adding the same amount of DTP@KA NPs, respectively. KA concentration in liposome solution was detected by UV–Vis–NIR instrument after being centrifuged.

### Experimental animal

All animal experiments and methods were ratified by the Animal Experimentation Ethics Committee of The Second Affiliated Hospital of Chongqing Medical University (IACUC-SAHCQMU-2023–0066). All procedures were conducted in accordance with the “Guiding Principles in the Care and Use of Animals” (China).

20–22 g Male C57BL/6 mice (2–3 mice per cage) were randomly grouped and raised under the standard environment (21 ± 0.5 °C, 55 ± 1% relative humidity and 12-light/12-dark cycle). The standard food and water were fed to mice for acclimatizing lasting at least 1 week,

### Experimental protocol

According to previous reports [17], 3.5% NAT was retrogradely injected into pancreatic duct to induce experimental SAP mice models. 25 or 50 mg/kg KA or saline intragastrically administrated. Additionally, 2.5 or 5 mg/kg DTM@KA NPs was injected into tail vein of mice for 24 h. The mice were sacrificed and the major organs and serum were collected for related experiments.

### Extraction of primary acinar cells (PAC)

1 mL of 200 U/mL collagenase solution was injected into the fresh pancreatic tissue along the catheter of the pancreatic head until possibly filled the entire pancreatic tissue, which was digested in water bath with 37 ℃ for 17 min. And then, 15 mL Hepes solution was added into the digested tissue to stop digestion. The solution next was filtered through 100 μm cell filter after being blew gently, and centrifuged for 2 min at 700 rpm. Finally, the precipitation was resuspend by Hepes to obtain 17 × 10^6^ PAC.

### Biosafety assessment

#### In vitro biosafety

CCK-8 kits was executed as the manufacturer's instructions to evaluating cell viability. The absorbance was read at 450 nm by SpectraMax M5 microplate reader (Molecular Devices, LLC, Sunnyvale, CA, USA) after KA (2.5 or 5 mg/kg) or DTP@KA (25 or 50 μg/kg) treatment for 24 h.

#### In vivo biosafety

The mice were sacrificed on 0, 7, 14, 28 days after KA or DTP@KA administration with different concentrations (25 or 50 μM), and the heart, liver, spleen, lung and kidney tissues were collected for hematoxylin and eosin (H&E) staining [18]. And then the morphological structure of tissues were observed under the microscope.

### Immunostaining

#### Immunohistochemistry (IHC) analysis

The SPN-9001 Histostain^™^-SP kits was conducted following product manual for IHC analysis. In brief, anti-TOM20 (1: 100), anti-STAT6 (1: 150), anti-BAX (1: 200), anti-Drp1 (1:200), Pink1 (1:200) and LC_3_-B (1:150) primary antibodies was used to incubate the tissue sections for overnight at 4 °C after repairing antigen, blocking endogenous peroxidase and serum; and then incubated with HRP-conjugated secondary antibody after being washed three times. Diaminobenzidine test kits was used to evaluate proteins’ distribution and expression. Next, the sections were stained with hematoxylin stain. Finally, ten representative sites of samples were randomly pictured for being semiquantitatively or quantitatively determined through two independent investigators in a blinded manner.

#### Immunofluorescent staining

The sections were incubated with anti-TOM20 (1: 100), anti-STAT6 (1: 150) Pink1 (1:200) and Parkin (1:150) at 4 ℃ for overnight, washed three times with PBS, and the anti-mouse or anti-rabbit antibodies were added for covering samples as the corresponding secondary antibodies. Finally, the slides were incubated by DAPI staining for 5 min at room temperature, and then were analyzed with a fuorescence microscope.

### Western blot

500 μl RAPA buffer containing cocktail inhibitors was added to lyse 50 mg pancreatic tissues, and its proteins was extracted by using the freeze grinder from Servicebio (KZ-III-F, Wuhan, China) following the manufacturer’s parameters. And then the lysate was placed on ice for 1 h, centrifuged at 12,000 g for 15 min and under 4 ℃, collected the supernatant and quantified with BCA assay kit (Beyotime, China). Next, SDS–polyacrylamide gel was used to load and separate 20 μg proteins, which next was electrophoretically transferred onto a PVDF membrane and blocked in 5% (w/v) nonfat dry milk for 1 h at 37 ℃. The membrane was incubated in the primary antibodies for overnight at 4 ℃. The secondary antibody was added to incubate membrane at 37 ℃ for 1 h, ECL test kits (Beyotime, China) was used to detect protein bands and be pictured or analyzed by Image Lab analysis software (Bio-Rad, California, USA).

### Statistical analysis

The data were tested by K-S (Kolmogorov–Smirnov) method for normality. Mean ± SEM was calculated for analyzing the data via Student’s t-test or one-way ANOVA analyses. All data were processed by SPSS 24.0 statistical software. *p* < 0.05 meant that the difference was statistically significant; ^*^p < 0.05, ^**^p < 0.01 and ^***^p < 0.001.

## Results

### Characterization

**Fig. 1:**
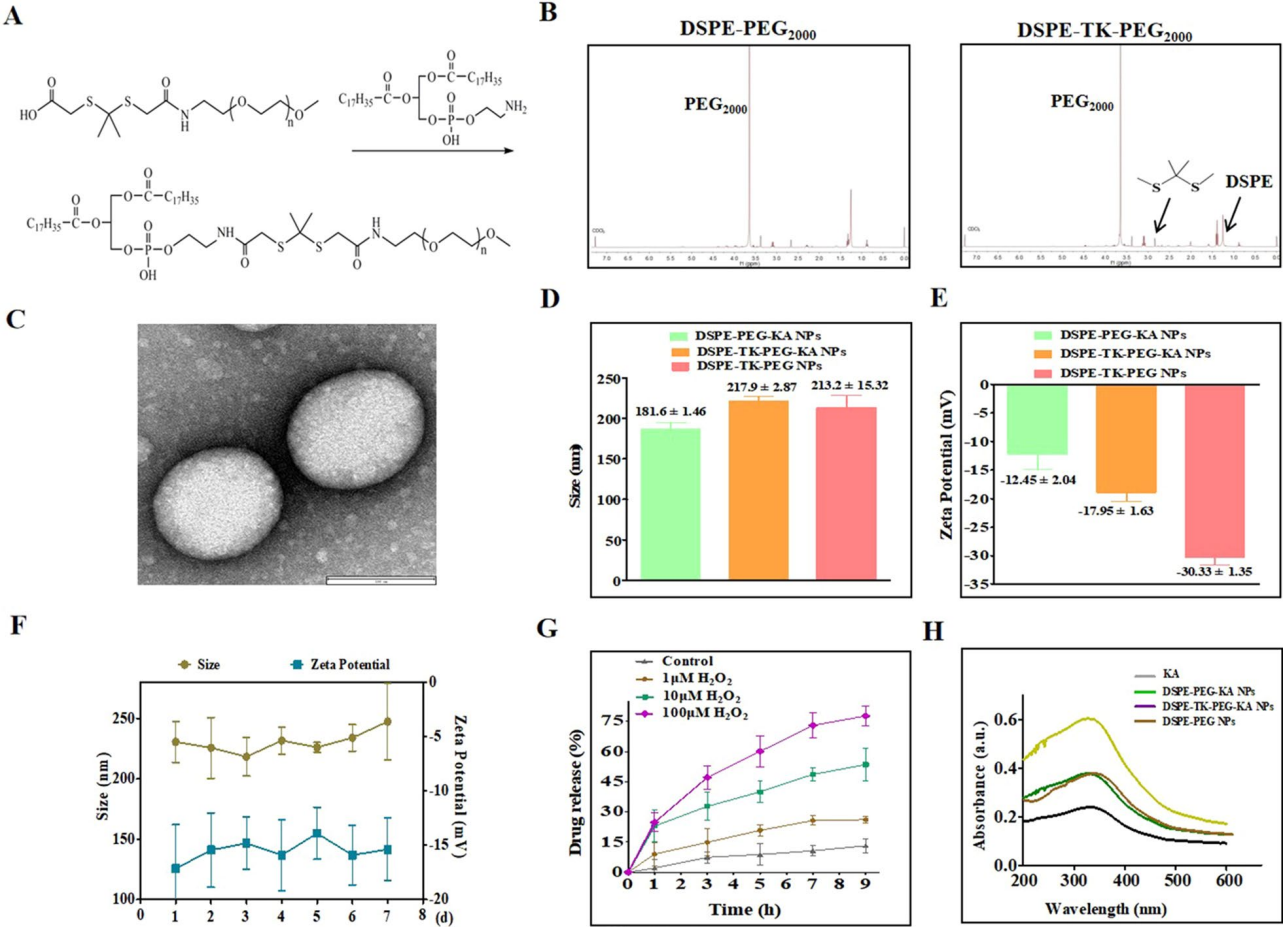
Characterization of lipidosome. **A**. A schematic diagram of the synthesis of DTP NPs. **B**. The ^1^H NMR spectra of DP (DSPE-PEG_2000_) and DTP (DSPE-TK-PEG_2000_). **C**. The representative TEM image of DTP@KA NPs. Scale bar = 100 nm. **D**. The average size. **E**. The average zeta potential. **F**. The stability of DTP@KA NPs in size and zeta potential for 7 days. **G**. The drug release curve of DTP@KA NPs stimulated by H_2_O_2_. **H**. The UV–Vis–NIR absorption spectra. Data represent the mean ± SEM of at least three independent experiments

Given the approximately 2% bioavailability and low absorption level of KA in rats [19], enhancing its systemic bioavailability and tissue delivery may well harness the benefit of KA as a therapeutic agent for SAP. As displayed in Scheme 1A, we designed a ROS-responsive nanodrugs featuring a TK bond inserted in DSPE-PEG double layer structure to delivery KA for SAP therapy. In this nanosystem, DTP (DSPEG-TK-PEG_2000_) was synthesized to deliver KA based on the pathological characteristics of SAP to maximize the targeting on pancreatic inflammatory injury sites and improve the bioavailability of KA (Fig. 1A). The structures of DP (DSPE-PEG_2000_) and DTP were detected by ^1^H-NMR, which verified the success of TK addition (Fig. 1B). Next, DTP was designed to load KA for overcoming its obstacles of low bioavailability. As shown in Fig. 1C, the TEM results showed a regular spherical morphology and uniform core/shell structure of DTP@KA NPs. The average diameters of DP@KA NPs, DTP@KA NPs and DTP NPs were 181.6 ± 1.46 nm, 217.9 ± 2.87 nm and 213.2 ± 15.32 nm, respectively (Fig. 1D). In addition, the zeta potentials were determined to be − 12.45 ± 2.04 mV, -17.95 ± 1.63 mV and − 30.33 ± 1.35 mV (Fig. 1E), respectively. Furthermore, the stability of DTP@KA NPs was measured for 7 consecutive days, and small fluctuations in size and zeta potentials within reasonable range was found (Fig. 1F). Besides, ROS triggered DTP@KA NPs’ release, and the release amounts was gradually increased with the stimulation of H_2_O_2_ from low to high concentrations (Fig. 1G). Moreover, the UV absorption curves of DTP@KA NPs were plotted by UV–Vis-NIR assay, and the encapsulation efficiency and loading efficiency were calculated to be 71.95 and 6.02%, respectively (Fig. 1H). These implies that DTP@KA NPs performs satisfactorily with suitable size, zeta potentials and stability, laying a well basis for in vivo application (Additional file 2: Table S1).

### Evaluation on safety profile, antioxidant capacity and pancreas tissues-targeting property of DTP@KA NPs

**Fig. 2:**
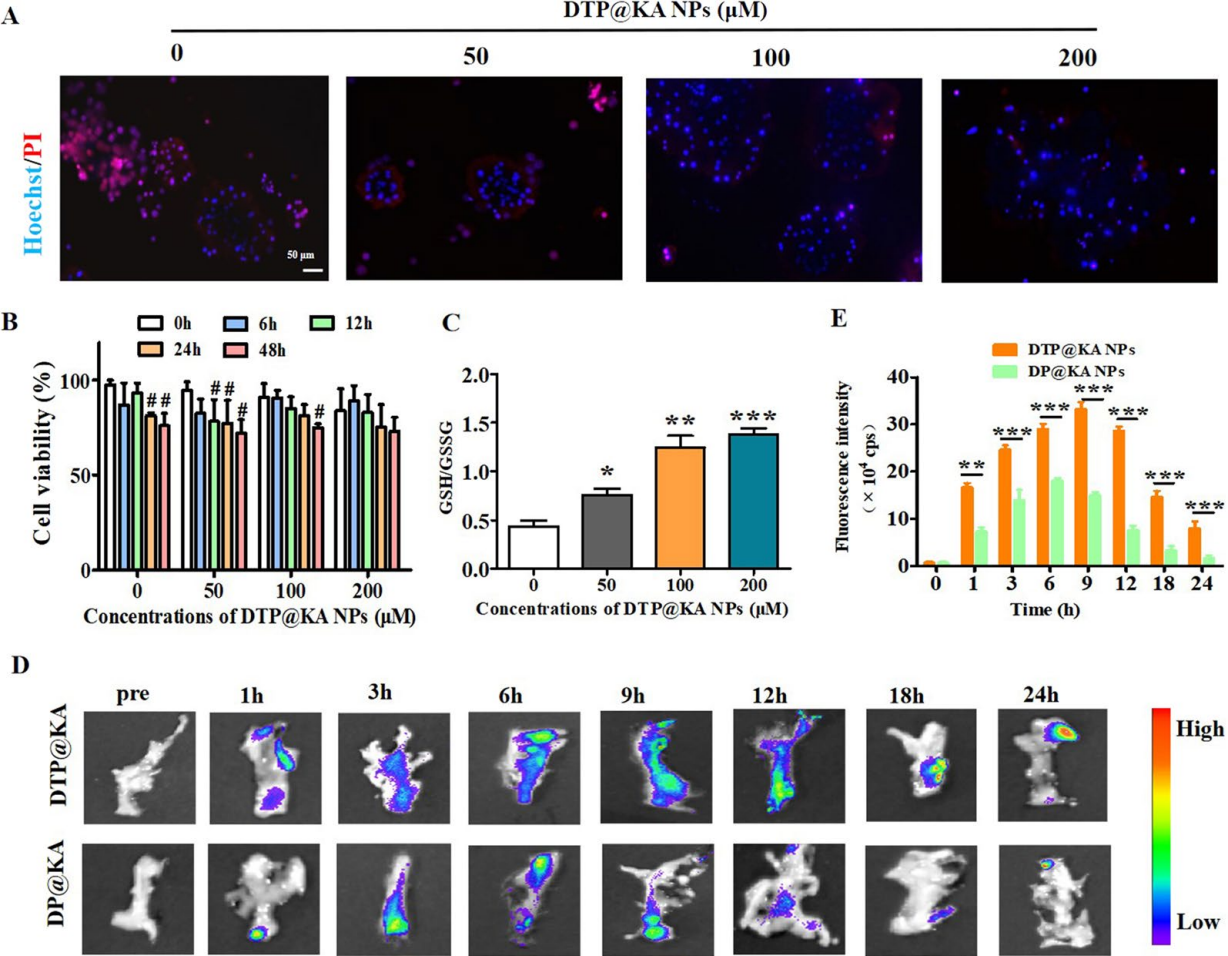
The antioxidation and pancreas-targeting property of DTP@KA NPs. **A**. Representative Hoechst33342/PI staining images of primary PAC after 100 μM H_2_O_2_ administration. Scar bar = 50 μm. **B** Viabilities of primary PAC co-incubated with different concentrations of DTP@KA NPs for 0 h, 6 h, 24 h and 48 h. **C** Glutathione (GSH)/oxidized glutathione (GSSG) ratio in primary PAC under DTP@KA NPs treatment with different concentrations. **D**, **E** In vitro DIR fuorescence images **D** and quantitative analysis **E** of pancreas from SAP model mice treated with DTP@KA NPs for 24 h. Data represent the mean ± SEM of at least three independent experiments; n = 5–8/group. Significance: ^#^*p* < 0.05 vs 0 h. ^*^ *p* < 0.05, ^**^ *p* < 0.01 and ^***^ *p* < 0.001 vs. the DP@KA NPs groups

Encouraged by KA as a natural antioxidant and DTP@KA NPs’ sensitive to ROS, we detected cell viability under a oxidative stress induced by 100 μM H_2_O_2_. Dead/live cell assays were detected by Hoechst 33342/PI staining to visually evaluate the antioxidant capacity of DTP@KA NPs under oxidative stress. As illustrated in Fig. 2A, DTP@KA NPs significantly inhibited PI intake into primary PAC in a concentration dependent manner.As shown in Fig. 2B, PAC without DTP@KA NPs treatments was significantly damaged by 100 μM H_2_O_2_ at 24 h and 48 h; and 50 μM DTP@KA NPs have no effect on PAC manifested by the obviously reduced cell viability after 12–48 h with 100 μM H_2_O_2_ stimulation; while 100 μM DTP@KA NPs distinctly inhibited PAC death damaged by 100 μM H_2_O_2_ before 48 h. Notably, 200 μM DTP@KA NPs significantly protect PAC injured by 100 μM H_2_O_2_ during 48 h_._ Glutathione (GSH) depletion is closely related to ROS accumulation, and promotes the occurrence of cell death, and ROS removal is accompanied by GSH oxidization into oxidized glutathione GSSG [20]. As displayed in Fig. 2C, the ratio of GSH/GSSG was increasing after DTP@KA NPs treatments in a concentration dependent manner (0, 50, 100 and 200 μM). Besides, we also determined the pancreas tissues-targeting properties of 200 μM DTP@KA NPs for evaluating its therapeutic potential in the experimental SAP. As revealed in Fig. 2D, E, DIR-labeled DTP@KA NPs in pancreas tissues was obviously increased more and lasted longer compared to 200 μM DP@KA NPs, implying that TK assists DTP@KA NPs in targeting injury section of pancreas. These results clearly indicate that DTP@KA NPs has a potential for reducing ROS accumulation in primary PAC, conducing to effectively inhibiting oxidative stress in primary PAC, and displays admirable targeting at damaged pancreas for a longer retainability.

**Fig. 3:**
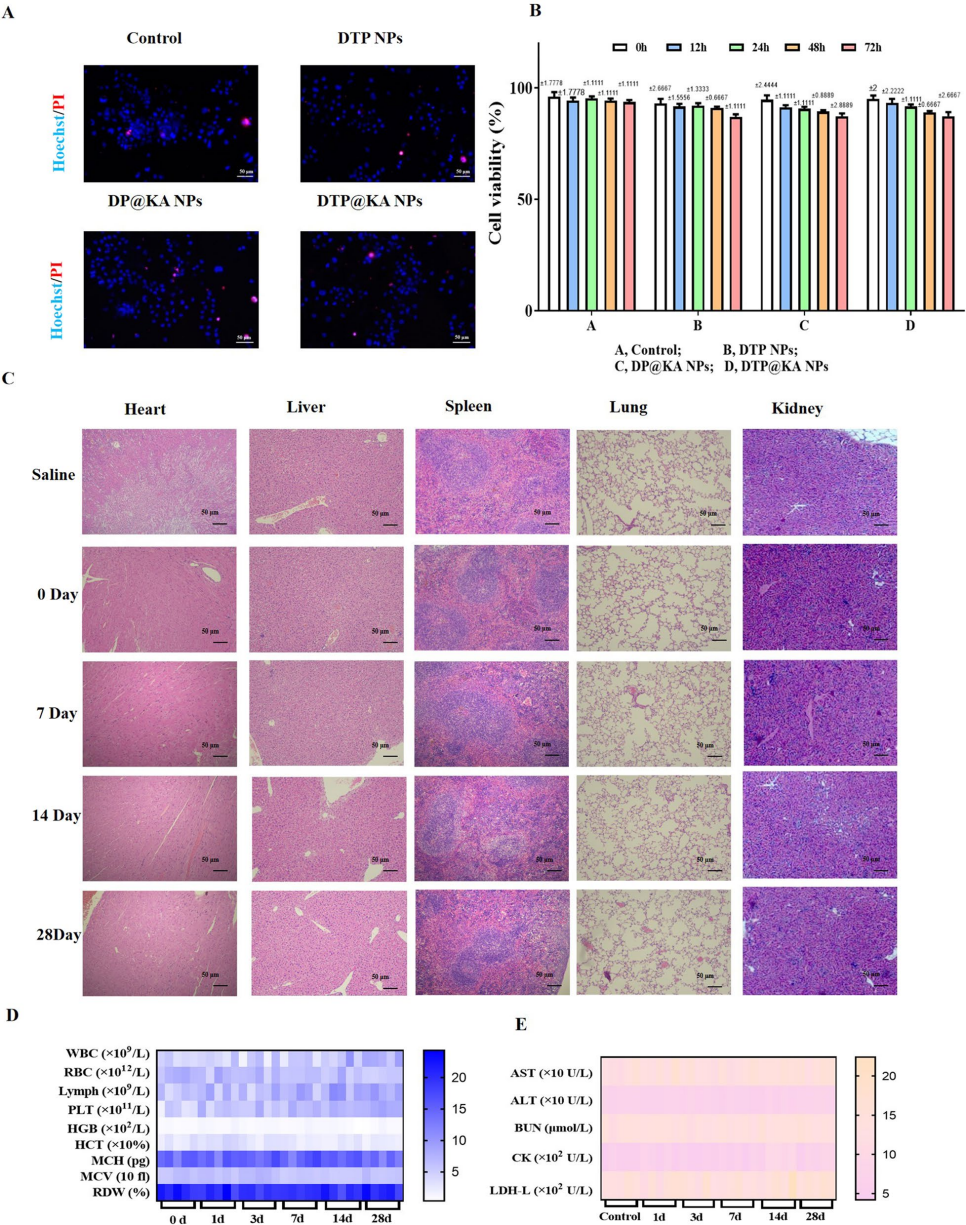
The safety profile of 200 μM DTP@KA NPs. **A**. Typical pictures of Hoechst33342/PI staining in primary PAC. Scar bar = 50 μm. **B** Cell viability of primary PAC. **C** Representative H&E staining images of heart, liver, spleen, lung and kidney after DTP@KA NPs administration. Scar bar = 50 μm. **D**, **E** Haematological indices.Data represent the mean ± SEM of at least three independent experiments and at least three sets of petri dishes; n = 5–8/group

In addition, drug safety directly affects the prospects of clinical transformation and advanced applications. The safety of 200 μM DTP@KA NPs in vivo and in vitro were investigated, respectively. As shown in Fig. 3A, NPs exhibited no effect on primary PAC viability, and hardly caused cell death during 72 h (Fig. 3B). Consistently, 200 μM DTP@KA NPs administration did not cause pathological damage to organs, including heart, liver, spleen, lung and kidney of mice, within 28 days compared to control group (Fig. 3C). Surprisingly, haematological indices of liver and kidney toxicity exhibited no variations after 200 μM DTP@KA NPs treatment (Fig. 3D, E). These results imply a good biocompatibility and application potential of 2 00 μM DTP@KA NPs. It all lays a promising foundation for the application of 200 μM DTP@KA NPs in SAP.

### DTP@KA NPs administration is beneficial in SAP

**Fig. 4:**
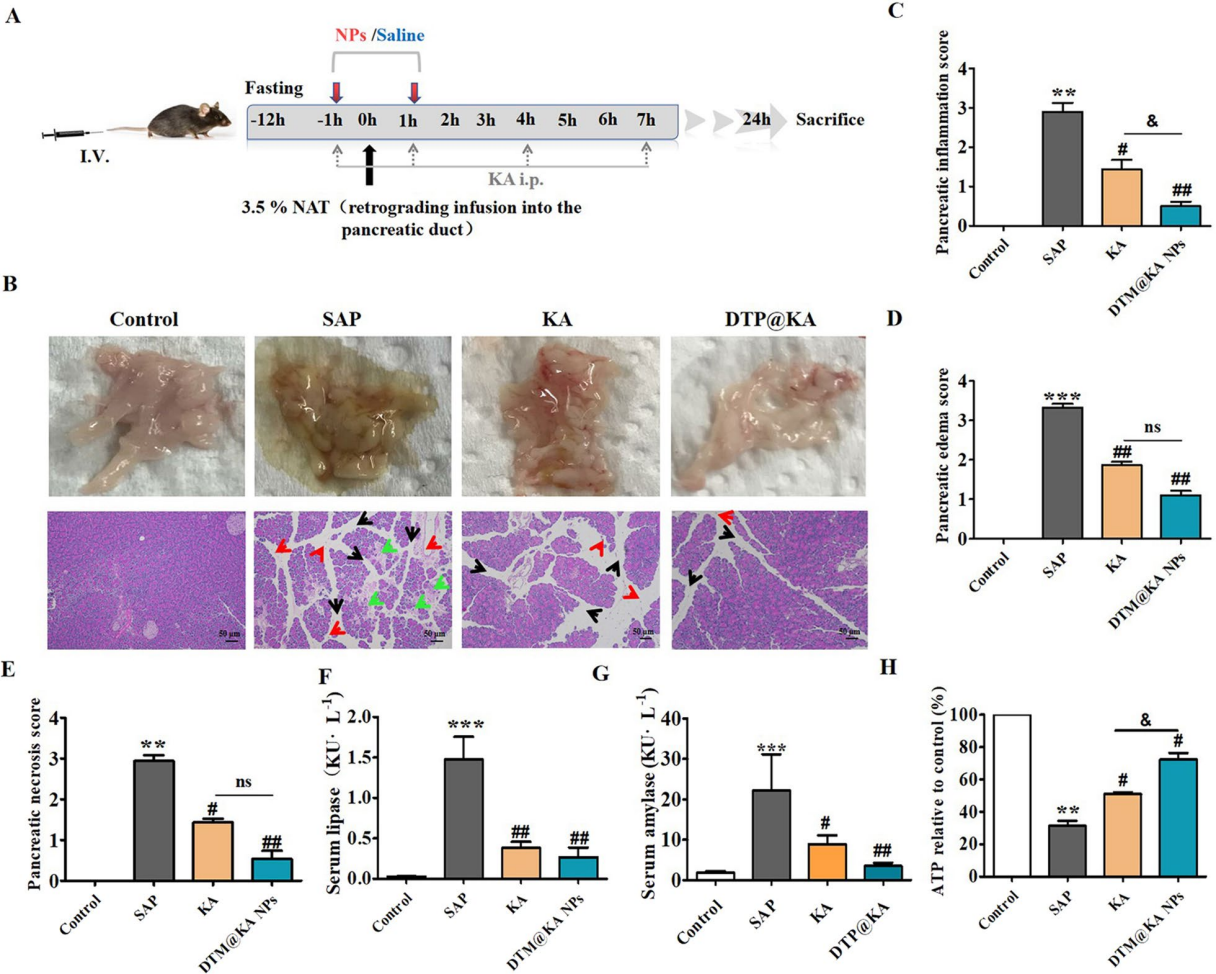
DTP@KA NPs was protective in experimental SAP. **A**. Schematic statement of experimental SAP and intervention protocols. **B**. Typical pictures of fresh pancreatic tissues and representative H&E images of pancreas. Scar bar = 50 μm. Red arrow, inflammation infiltration; black arrow, edema; green arrow, necrosis. **C**. Inflammatory injury scores of pancreatic tissues. **D**. Edema scores of pancreatic tissues. **E**. Necrosis score of pancreatic tissues. **F**. Serum lipase levels. **G**. Serum amylase levels. **H** ATP contents. Data represent the mean ± SEM of at least three independent experiments; n = 5–8/group. Significance: ^**^ *p* < 0.01 and ^***^ *p* < 0.001 vs. the control group; ^#^ *p* < 0.05 and ^##^ *p* < 0.01 vs. the SAP model group; ^&^ *p* < 0.05 vs. the KA group

KA, a natural antioxidant and anti-inflammatory agent, has great potential in alleviating pancreatitis [21]. 50 mg/kg KA or 5 mg/kg DTP@KA NPs was chosen to be administrated 1 h before and after the injection of NAT (Additional file 1: Fig S1 and Fig. 4A), respectively, to investigate whether DTP@KA NPs was more effective in experimental SAP. KA and DTP@KA NPs improved the pathological status of pancreatic inflammatory infiltration (Fig. 4B, C), edema (Fig. 4B and D), necrosis (Fig. 4B and E), serum lipase (Fig. 4F) and amylase (Fig. 4G) induced by NAT. Especially, DTP@KA NPs more effectively inhibited the inflammatory response of pancreas than KA administration (Fig. 4C); moreover, DTP@KA NPs exhibited a stronger effect on reducing ATP depletion compared to KA (Fig. 4H). Hence, it would be expected that the administration of DTP@KA NPs will greatly suppress inflammatory injury of SAP.

### DTP@KA NPs inhibits mitochondrial dysfunction and oxidative stress

**Fig. 5:**
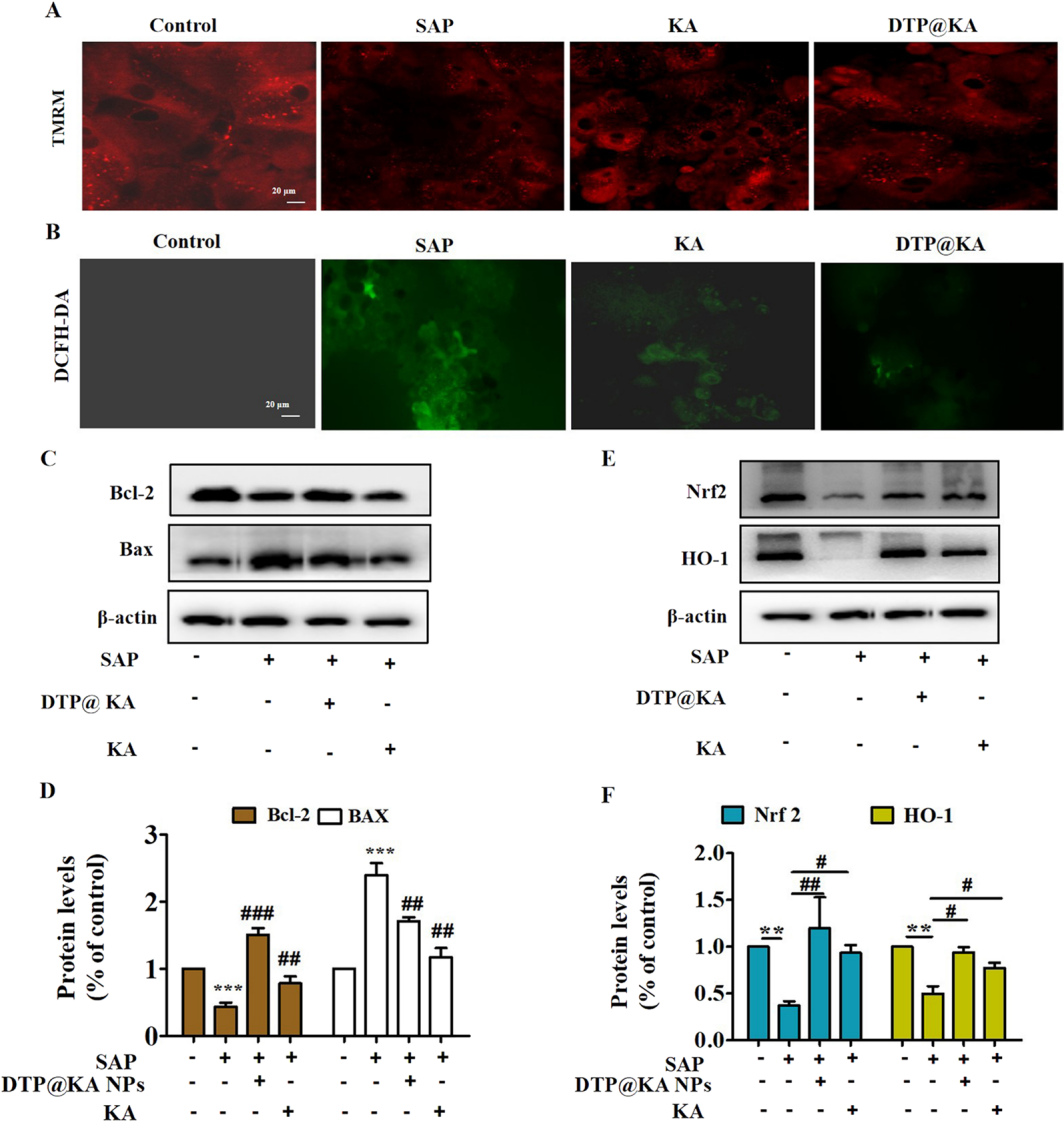
DTP@KA NPs improved mitochondrial injury. **A**. Typical pictures of TMRM staining images of pancreas. Scar bar = 20 μm. **B**. Representative images of DCFH-DA staining in pancreatic tissues. Scar bar = 20 μm. **C**. Protein expressions of BAX and BCL-2. **D**. Quantitative analysis of BAX and BCL-2 protein expressions. **E**. Protein expressions of Nrf2 and HO-1. **F**. Quantitative analysis of Nrf2 and HO-1 protein expressions. Data represent the mean ± SEM of at least three independent experiments; n = 5–8/group. Significance: ^**^ *p* < 0.01 and ^***^p < 0.001 vs. control group; ^#^ *p* < 0.05, ^##^ *p* < 0.01 and ^###^ *p* < 0.001 vs. the SAP model group

As indicated in the above findings, DTP@KA NPs particularly inhibited inflammation and ATP depletion, which is closely related to mitochondria function. We further assessed mitochondrial homeostasis. Here, TMRM staining was performed for assaying DTP@KA NPs’ effect on mitochondrial membrane potential (MMP) to estimate mitochondrial integrity. As shown in Fig. 5A, DTP@KA NPs inhibited the loss of MMP with increased TMRM fluorescence intensity. Mitochondrial function is inextricably linked to ROS generation and apoptosis process. DTP@KA NPs reduced ROS accumulation in pancreas with decreased DCFH-DA fluorescence intensity (Fig. 5B), suggesting DTP@KA NPs’ improvement on mitochondrial damage in pancreas. In addition, reduced pancreatic BAX expression and enhanced BCL-2 expression also was investigated for displaying DTP@KA NPs’ efficiency on inhibiting mitochondria in apoptosis (Fig. 5C, D). Moreover, DTP@KA NPs exerted anti-oxidative activity via increasing Nrf2 and HO-1 expressions in pancreas tissues (Fig. 5E, F). These manifest that DTP@KA NPs shows a terrific protection effect on mitochondrial damage, and it is possibly through alleviating mitochondrial dysfunction to suppress oxidative stress.

### DTP@KA NPs improves mitochondrial fission and mitophagy via activating TOM20-STAT6 signaling

**Fig. 6:**
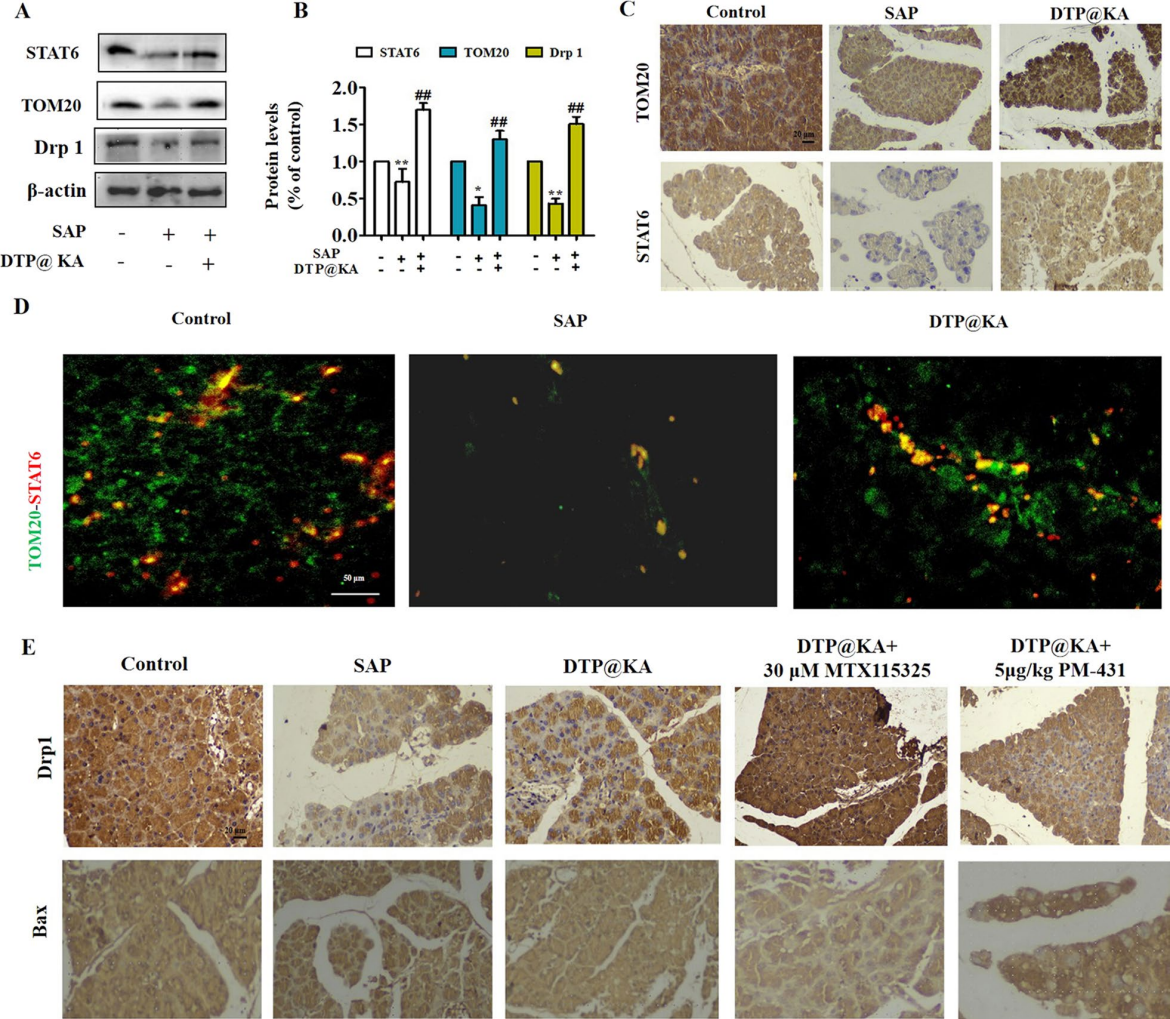
DTP@KA NPs improved mitochondrial fission by activating TOM20-STAT6 signaling pathway. **A**. Protein expressions of STAT6, TOM20 and Drp1 in pancreas lysate. **B**. Quantitative analysis of STAT6, TOM20 and Drp1 protein expressions. **C**. Distributions and expressions of STAT6 and TOM20 in pancreas tissues. Scar bar = 20 μm. **D**. Co-localization and expressions of TOM20 and STAT6 with IF staining in pancreas tissues. Scar bar = 50 μm. **E**. IHC staining of Drp1 and Bax in pancreas tissues. Scar bar = 20 μm. Data represent the mean ± SEM of at least three independent experiments; n = 5–8/group. Significance: ^*^ *p* < 0.05 and ^**^p < 0.01 vs. the control group; ^#^ *p* < 0.05, ^##^ *p* < 0.01 vs. the SAP model group

Based on the protective efficacy of DTP@KA NPs on mitochondrial damage in experimental SAP, the possible mechanism was further explored. As shown in Fig. 6A, B, SAP resulted in inhibition of STAT6 expression and TOM20 levels, while DTP@KA NPs significantly up-regulated their expressions and improved the inadequate mitochondrial fission by enhancing Drp1 expression in pancreas tissues. Similarly, IHC results also was proved that DTP@KA NPs promoted the distributions and expressions of STAT6 and TOM20 in pancreas tissues (Fig. 6C). Interestingly, STAT6 was found to be connected with TOM20 (Fig. 6D), suggesting that STAT6-regulated transcription of mitochondrial precursor proteins or transduction of downstream signaling molecular may be assisted by TOM20 activation. Next, MTX115325 (agonist of TOM20) and PM-431 (inhibitor of STAT6) was administrated respectively in SAP for further investigation on mitochondrial function. Importantly, an enhanced Drp1 and declined Bax distribution levels was found after activating TOM20, while reversed by inhibiting STAT6 (Fig. 6E). These demonstrate that DTP@KA NPs exhibits an improvement activity in mitochondrial possibly by enhancing mitochondrial fission via promoting the interaction and activation between STAT6 and TOM20.

**Fig. 7:**
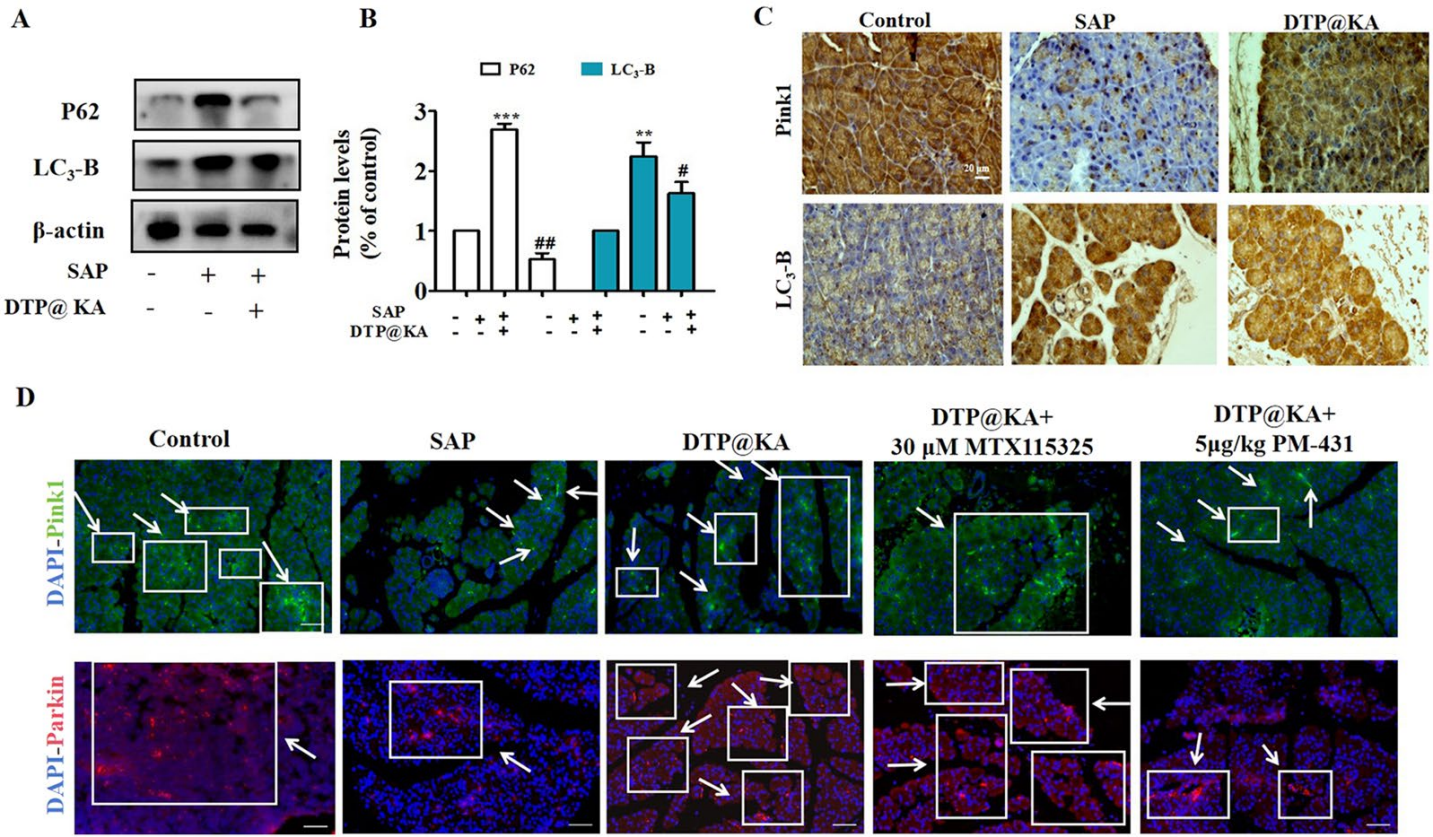
DTP@KA NPs promoted mitophagy by TOM20-STAT6 signaling pathway. **A**. Protein expressions of PINK1, Parkin, P62 and LC3-II in pancreas lysate. **B**. Quantitative analysis of PINK1, Parkin, P62 and LC3-II protein expressions. **C**. Distributions and expressions of PINK1 and Parkin in pancreas tissues. **D**. IF staining of PINK1 and Parkin in pancreas tissues. Green fluorescence, Pink1 expression; Red fluorescence, Parkin expression.Data represent the mean ± SEM of at least three independent experiments; n = 5–8/group. Significance: ^**^ *p* < 0.01 and ^***^p < 0.001 vs. the control group; ^#^ *p* < 0.05, ^##^ *p* < 0.01 vs. the SAP model group

Inadequate mitochondrial fission leads to the failure of damaged mitochondria to split out in time, hindering the clearance of damaged mitochondria by autophagy, which aggravates mitochondrial damage with accumulation [22]. To investigate the influence of SAP on mitophagy in TOM20-STAT6-regulating mitochondrial dysfunction, specific activator of TOM20 and inhibitor for STAT6 were applied respectively. The addition of MTX115325 and PM-431 enhanced DTP@KA NPs’ effect on up-regulating the protein expressions of PINK1 and Parkin as well as down-regulating LC_3_-B expression in pancreas tissues, indicating that an increased mitophagy may contribute to cleaning injured mitochondria via the promoted lysosomal degradation with decreased P62(Fig. 7A, B). Meanwhile, IHC and IF results also manifested an enhanced distributions and expressions of PINK1 and decreased LC_3_-B expression after DTP@KA NPs administration (Fig. 7C), suggesting that DTP@KA NPs promoted mitophagy by enhancing Pink 1 activity accompanying by reduced autophagosomes. Besides, it was proved that DTP@KA NPs could be further increased mitophagy by activating TOM20 and reduced it by suppressing STAT6 activation in experimental SAP (Fig. 7D).

Thus, DTP@KA NPs administration protects against SAP possibly via restoring mitochondrial fission and mitophagy with activation of the TOM20-STAT6 signaling pathway.

### DTP@KA NPs inhibits inflammation and organs dysfunction

**Fig. 8:**
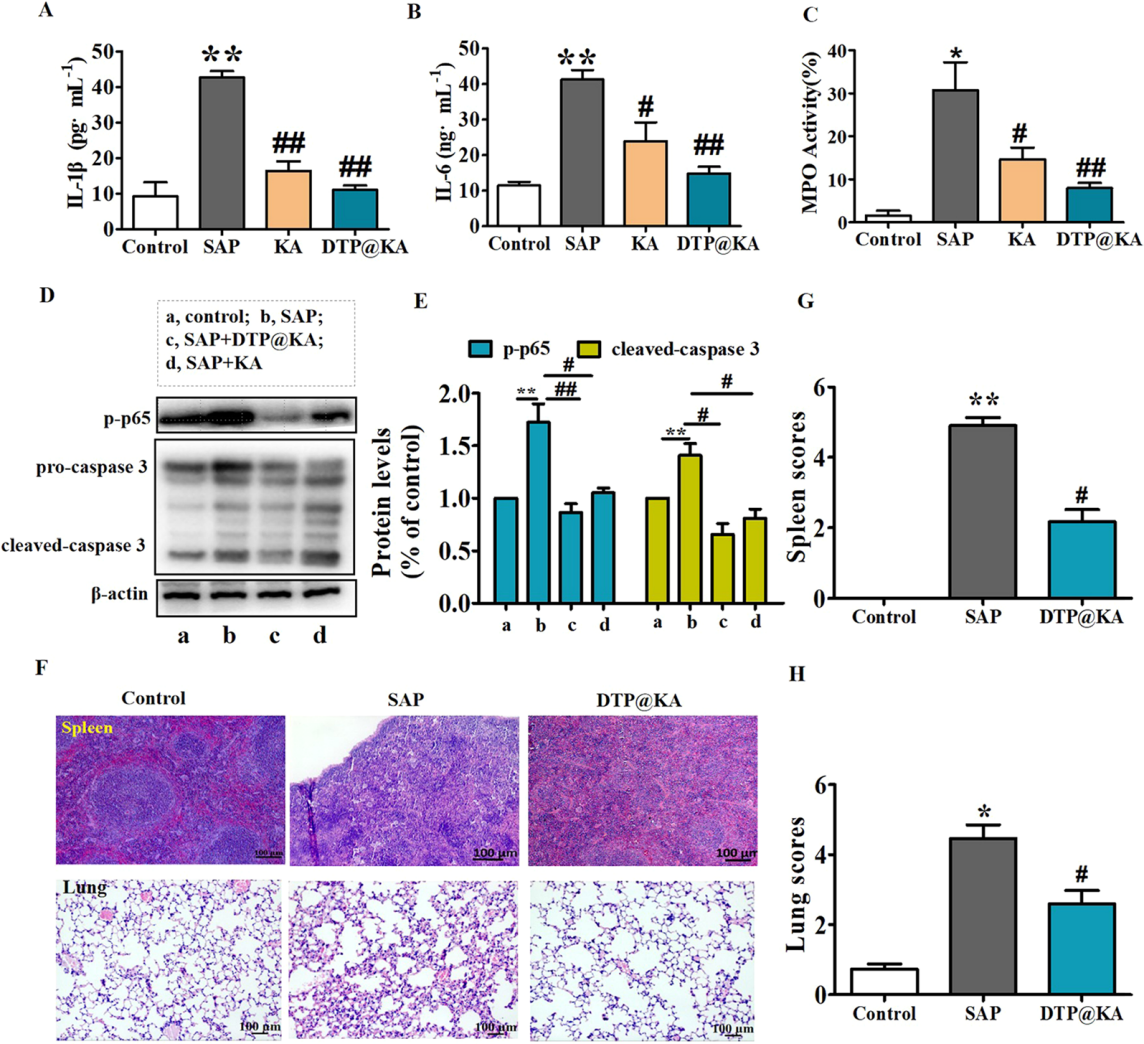
DTP@KA NPs alleviated inflammation and multiple organ damage. **A**–**C**. Activity assay IL-1β (**A**), IL-6 (**B**) and MPO (**C**) in pancreas. **D**. Protein expressions of p-P65 and caspase3. **E**. Quantitative analysis of p-P65 and caspase3 protein expressions. **F**. Representative HE images of spleen and lung tissues. Scar bar = 100 μm. **G**. Spleen scores. **H**. Lung scores. Data represent the mean ± SEM of at least three independent experiments; n = 5–8/group. Significance: ^*^ *p* < 0.05 and ^**^p < 0.01 vs. the control group; ^#^ *p* < 0.05 and ^##^ *p* < 0.01 vs. the SAP model group

Mitochondrial dysfunction exacerbates inflammation and apoptosis of cells [23]. Lower IL-1β (Fig. 8A) and IL-6 (Fig. 8B) in pancreas was detected after DTP@KA NPs application compared to KA administration. Inflammatory spread is the most important factor to aggravate pancreatitis [24]. As shown in Fig. 8C, DTP@KA NPs displayed a stronger effect on the inhibition of inflammatory cell infiltration, manifested by lower MPO activity in pancreas tissues compared to KA. Meanwhile, large amounts of inflammation promotes cell death [25]. We found that the expression of p-p65 and cleaved-caspase 3 were observed a reduction in pancreas tissues after DTP@KA NPs administration (Fig. 8D, E). Furthermore, DTP@KA NPs apparently inhibited inflammatory cell infiltration in other tissues, such as spleen and lung tissues (Fig. 8F). As illustrated in Fig. 8G, H, DTP@KA NPs also can hinder SAP-related spleen injury and lung damage. These indicate that DTP@KA NPs not only inhibits the spread of inflammation to exacerbate pancreas injury with inhibited apoptosis, but also suppress damage to other organs. Taken together, these findings make it possible for DTP@KA NPs to be applied in clinical practice.

## Discussion

Regulation of mitochondrial function is critical to the progression of SAP [26]. Mitochondrial processes including dynamics, biogenesis, transcription-respiration, apoptosis and mitopahgy was mainly regulated by mitochondria-localized nuclear transcription factors; STAT6 has been reported as a mitochondrial protein in outer membrane of mitochondria for regulating mitochondrial dynamics [27]. Here, the disrupted mitochondrial dynamics with inadequate fission was detected in SAP model mice, accompanied by ATP depletion, ROS generation, mitochondrial apoptosis and mitophagy impairment, which is likely to impel SAP progression with multiple organ failures. Significantly, reduced STAT6 hinders the normal transcription of mitochondrial proteins and induces mitochondrial dysfunction, which reduced by the inhibited TOM20 interaction in SAP. Thus, targeting STAT6-TOM20-regulated mitochondria function exhibits a potential therapeutic approach for SAP.

KA, a flavonoid from TCM, fruits and vegetables, was proved to inhibit oxidation, inflammation and cell death via protecting mitochondria, while its efficacy limited by the low water solubility and poor bioavailability [6]. Nano-formulated water soluble KA has been widely used to improve its defects [28]. SAP is closely linked to systemic inflammation induced by ROS accumulation [29]. TK bond, responsive to ROS, is widely applied in acute inflammation [30]. Here, we engaged in constructing an liposome including TK bond with excellent biocompatibility, solubilizing property and targeting to delivery KA and enhance its function on anti-oxidation and restoration of mitochondrial function, namely DTM@KA NPs. Consistent with current studies’ findings in spesis and acute inflammatory disease [31], DTM@KA NPs treatment decreased inflammation and maintained organs function in SAP. DTM@KA NPs’ perfect efficacy indicated its great potential for clinical application in SAP or systemic inflammation. It is known that KA as an natural antioxidant not only scavenges ROS accumulation but also strongly targeted damaged mitochondria [32]. Studies have shown that an accumulation of impaired mitochondria is common to SAP [33]. It hints that strategies for eliminating such dysfunctional mitochondria benefits from DTM@KA NPs.

Notably, DTM@KA NPs shown an adequate removal of damaged factors via enhancing fission and mitophagy, which is considered to be a important mechanism for hindering the development of SAP. Consistently, STAT6-TOM20-mediated mitochondria function we proved is an vital pathway for SAP inflammation. Here, we explored the underlying mechanism on relationship between mitochondrial dynamics and STAT6-TOM20 interaction influenced by DTM@KA NPs. In this regard, DTM@KA NPs was proved to up-regulate the expression and interaction of STAT6-TOM20, especially showed an enhancement after activating TOM20 or a decrease when inhibiting STAT6 activity, which is in accordance with the above assumption. In addition, insufficient fission seriously hinders timely clearance of mitochondria; and Drp1 is well known as an indispensable regulator for mitochondrial fission [34]. Significantly, the interaction of STAT6-TOM20 activated by DTM@KA NPs accelerates Drp1-dependent mitochondrial fission. More importantly, STAT6-TOM20 signaling also improving Parkin/Pink1-dependent mitophagy, which is crucial to diminishing mitochondrial accumulation for DTM@KA NPs’ protection against SAP. Therefore, DTM@KA NPs lowers the activation of an inflammatory response possibly via activating STAT6-TOM20/Drp1-regulating mitochondrial fission, and then enhanced mitophagy removed more damaged mitochondria to inhibit SAP-related injuries.

## Conclusion

Generally, we successfully constructed a mitochondria damage-related ROS-responsive nanoparticles (DTM@KA NPs) based on KA for intervening the redox homeostasis and mitochondria dysfunction in experimental SAP. In this nanosystem, DTM NPs nanoparticle was designed to response to mitochondrial disorders-induce oxidative stress and cell dyshomeostasis, the TK bond was used as a target at inflammation- and ROS-damaged areas in pancreas. Meanwhile, KA as an natural antioxidant and anti-inflammatory drugs was utilized to exhibit synergistic antioxidant and anti-inflammatory effects with TK, which contributes to improving mitochondrial homeostasis and redox homeostasis, and then inhibit SAP-related pancreas and other organs injury. Additionally, DTM@KA NPs also mitigated ROS accumulation, mitochondrial fission and mitophagy via activating STAT6 assisted by interacting with TOM20, which reduced accumulation of damaged mitochondria and possibly inhibited mitochondrial apoptosis and inflammation spread. Most importantly, DTM@KA NPs not only improved pancreatic damage but also prevented multiple organs failures in SAP. Besides, DTM@KA NPs was proved to possess a favorable security and validity, which enhances the possibility of clinical application. Overall, our work proposes a possibility for mitochondrial function-related anti-inflammation therapy with unbalanced intracellular redox homeostasis via regulating TOM20-STAT6-Drp1-mitophagy pathway, potentially offering feasible strategies for combination therapy of SAP.

### Supplementary Information

**Additional file 1: Figure S1.** 50 mg/kg KA or 5 mg/kg DTP@KA NPs was more protective in experimental SAP. (A). Typical pictures of fresh pancreatic tissues and representative H&E images of pancreas. Scar bar = 50 μm. (B). Pancreas scores. Data represent the mean ± SEM of at least three independent experiments; n=5-8/group. Significance: ** p < 0.01 vs. the control group; # p < 0.05 and ## p < 0.01 vs. the SAP model group; & p < 0.05 vs. the 50 mg/kg KA group. **Figure S2.** Bar chart split in figure3D. **Figure S3.** Liver and kidney function indexes.

**Additional file 2.** Blood routine examination.

## Data Availability

The additional data relevant to this study could be requested and obtained form the corresponding author without the violation of participant confidentiality.
