# Supplementary material for: The mitochondria-targeted Kaempferol nanoparticle ameliorates severe acute pancreatitis
Source: J Nanobiotechnology. 2024 Apr 3;22:148. doi: 10.1186/s12951-024-02439-y (PMC10993609; doi:10.1186/s12951-024-02439-y)
Supplement: Supplementary file 1 — Additional file 1: Figure S1. 50 mg/kg KA or 5 mg/kg DTP@KA NPs was more protective in experimental SAP. (A). Typical pictures of fresh pancreatic tissues and representative H&E images of pancreas. Scar bar = 50 μm. (B). Pancreas scores. Data represent the mean ± SEM of at least three independent experiments; n=5-8/group. Significance: ** p < 0.01 vs. the control group; # p < 0.05 and ## p < 0.01 vs. the SAP model group; & p < 0.05 vs. the 50 mg/kg KA group. Figure S2. Bar chart split in figure3D. Figure S3. Liver and kidney function indexes. [file 12951_2024_2439_MOESM1_ESM.docx]

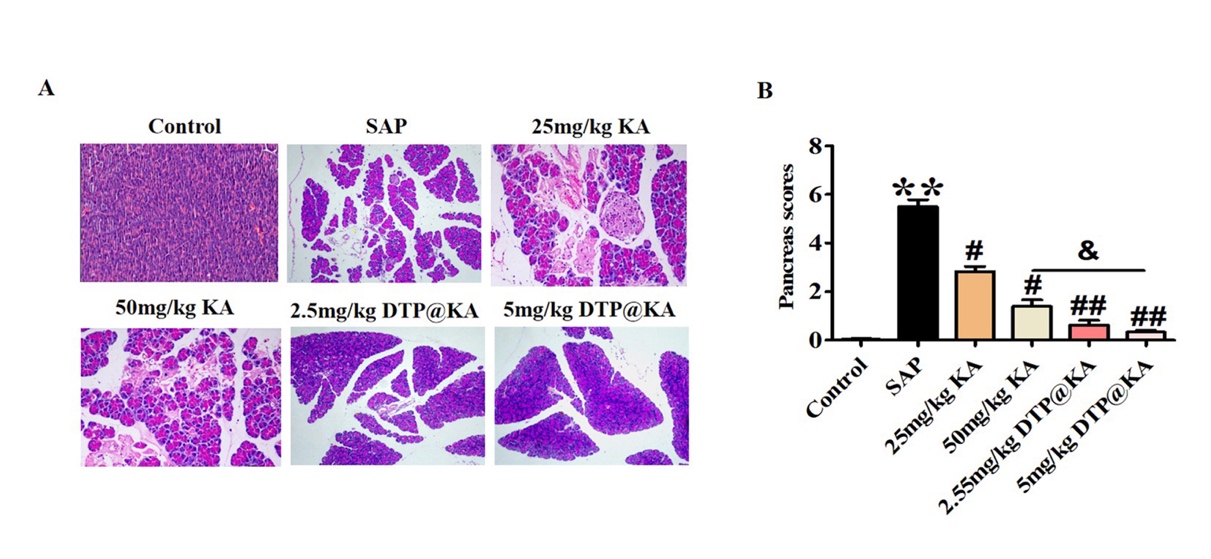


**Supplementary Figure 1**. 50 mg/kg KA or 5 mg/kg DTP@KA NPs was more protective in experimental SAP. (A). Typical pictures of fresh pancreatic tissues and representative H&E images of pancreas. Scar bar = 50 μm. (B). Pancreas scores. Data represent the mean ± SEM of at least three independent experiments; n=5-8/group. Significance: ^**^ *p* < 0.01 vs. the control group; ^#^ *p* < 0.05 and ^##^ *p* < 0.01 vs. the SAP model group; ^&^ *p* < 0.05 vs. the 50 mg/kg KA group.


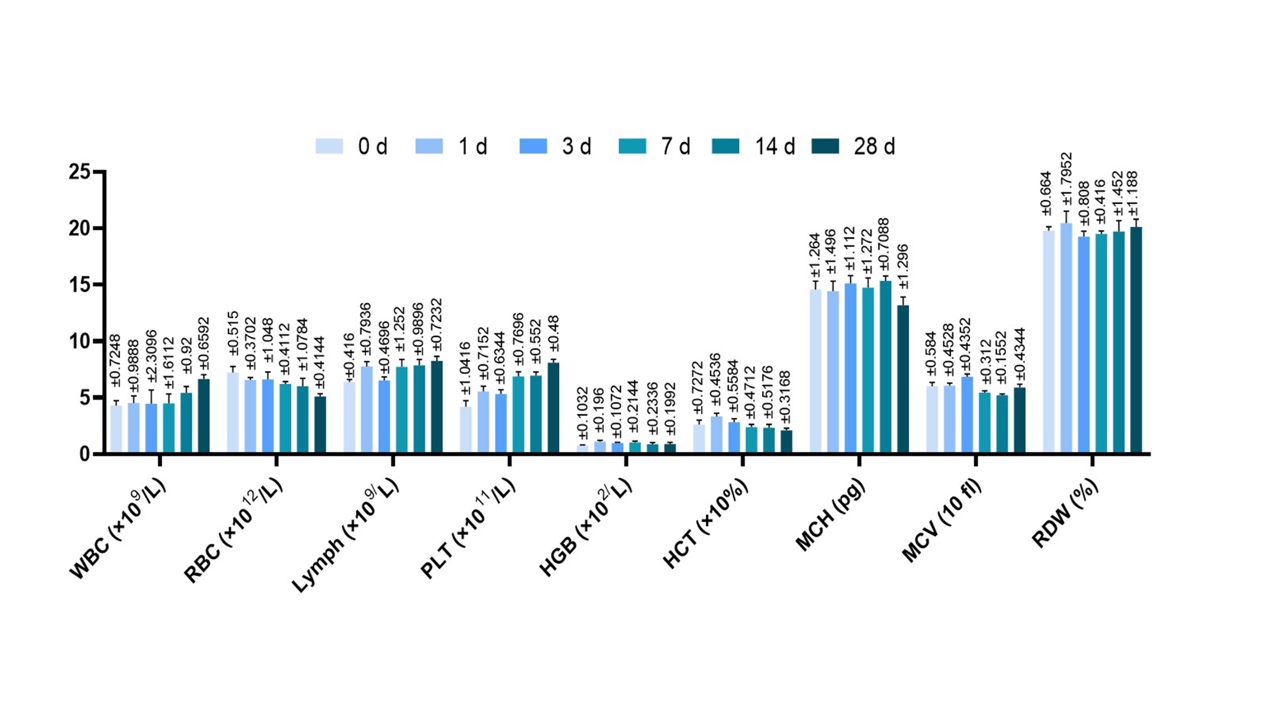


**Supplementary Figure 2.** Bar chart split in figure3D


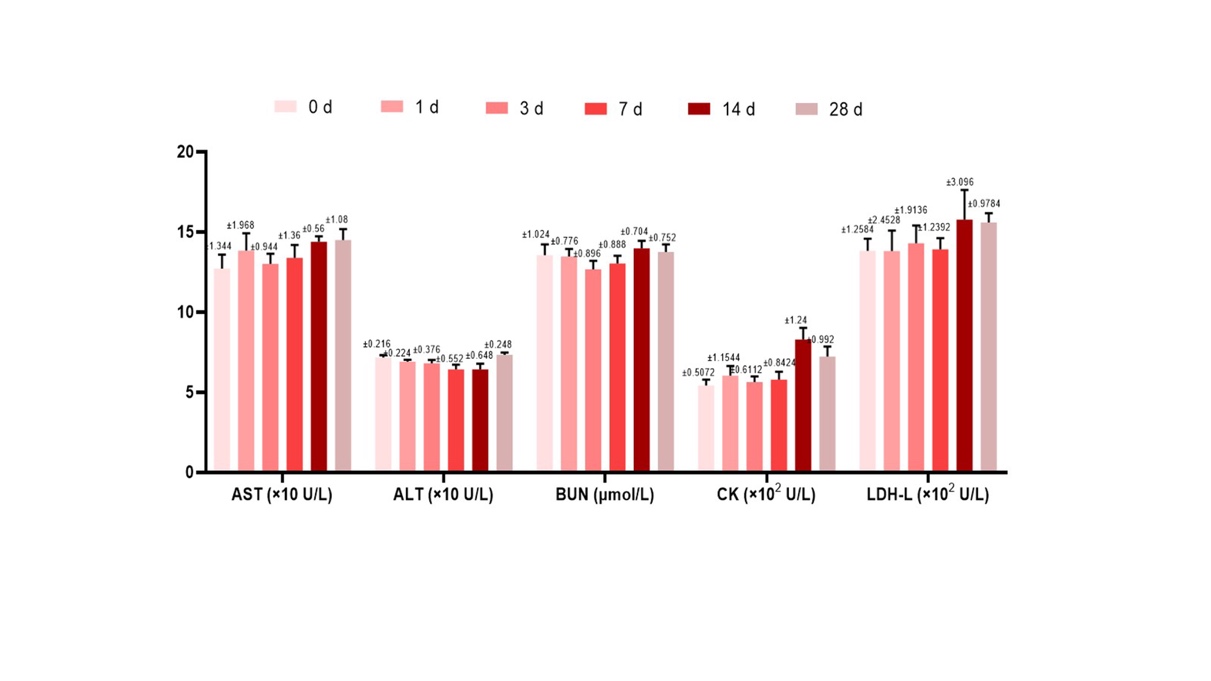


**Supplementary Figure 3.** Bar chart split in figure3E
